# Racial and Ethnic Minority Pregnant Patients with Low-Income Experiences of Perinatal Care: A Scoping Review

**DOI:** 10.1089/heq.2021.0017

**Published:** 2021-09-03

**Authors:** Danielle Wishart, Cindy Cruz Alvarez, Carmenisha Ward, Sankirtana Danner, Catherine A. O'Brian, Melissa Simon

**Affiliations:** ^1^ Keck School of Medicine, University of Southern California, Los Angeles, California, USA.; ^2^ Center for Health Equity Transformation, Northwestern University Feinberg School of Medicine, Chicago, Illinois, USA.; ^3^ Stritch School of Medicine, Loyola University Chicago, Maywood, Illinois, USA.; ^4^ College of Medicine, University of Illinois at Chicago, Chicago, Illinois, USA.; ^5^ Department of Obstetrics and Gynecology, Northwestern University, Chicago, Illinois, USA.

**Keywords:** perinatal care, health equity, maternal mortality, maternal morbidity

## Abstract

**Purpose:** The maternal mortality ratio for the United States (US) has consistently risen over recent decades. This mortality is especially pronounced within minority populations who experience a maternal mortality and morbidity rate that are much higher than their non-Hispanic white counterparts. Qualitative data are critical in gaining true insight from minority pregnant and postpartum persons. Such data should serve as the basis for building interventions and programs that seek to eradicate perinatal inequities. This review examines the qualitative literature on racial and ethnic minority pregnant patients with low income and their experiences during perinatal care (PNC) to identify recurrent themes that can be addressed through targeted interventions.

**Methods:** PubMed, CINAHL, and Web of Science databases were searched for qualitative studies on racial and ethnic minority pregnant patients with low income and their experiences during PNC. Twenty-two articles were included for analysis. Thematic synthesis was performed to identify categories and recurring themes in each article.

**Results:** Five major categories were identified as consistent experiences of pregnant patients with PNC clinicians: support, education, connection, communication, and trust. Of these, clinician support was the most consistently coded category. Eighteen of the 23 articles discussed tangible support patients had received from their clinicians, such as care coordination and referrals to support services. The second most coded category was education, which was represented in 16 articles. Education was mostly represented negatively as lack of adequate perinatal care education given during the perinatal period. Finally, the categories of connection, communication, and trust were represented by 18, 17, and 17 articles, respectively.

**Conclusions:** These qualitative studies provided specific examples of what racial and ethnic minority pregnant patients with low income deemed positive and negative during the perinatal period and outline ways that these experiences can be improved. Future studies can take the experiences reported in this review to help inform interventions to improve patient experiences and health outcomes that minority persons face in the perinatal period.

## Introduction

Over the past 20 years, worldwide efforts to improve conditions for pregnant patients have reduced the global maternal mortality ratio (MMR) by 38%.^1^ This reduction in mortality is unfortunately not reflected in the United States (US). With an MMR of 19 deaths per 100,000 live births in 2017, the U.S. rate was twice as high as high-income countries like Canada (MMR=10) and the United Kingdom (MMR=7).^1^ In fact, the MMR in the United States has increased since 1987, when the rate was 7.2.^2,3^

Racial breakdown of maternal mortality rates is alarming. According to the Centers for Disease Control and Prevention, individuals categorized as non-Hispanic black (NHB) are over 2.5 times more likely to experience a pregnancy-related death than individuals described as non-Hispanic white (NHW).^2^ In addition, Hispanic women experience severe maternal morbidity at a rate of more than two times their non-Hispanic white counterparts.^4^ Nonbiological factors such as socioeconomic status, social factors, and racism have been shown by the literature to impact a person's health.^5–8^ While the literature has shown certain biological causes of maternal mortality,^3,9^ data highlighting the social, economic, and structural factors that impact these biological causes of maternal mortality and morbidity are starting to emerge.^10,11^

The maternity care model, which depicts the sociocultural environment as impacting the outcomes of the maternal-child dyad throughout pregnancy, birth, and postpartum, is a conceptual framework that guides this article.^12^ Structural inequities and biases, such as sexism, racism, and classism, shape U.S. culture and intersect with all levels of the model. These structural equities are born out in everyday problems that minority pregnant patients must overcome, such as lack of access to affordable and safe housing, transportation, safe and well-paying job opportunities, quality and affordable health care, and more.^12^ These inequities are also tied to mistrust in the system from the decades of forced experimentation and abuse of minorities in medicine,^13,14^ including obstetrician and gynecologist, Dr. Marion Sims, who conducted extensive unanesthetized experimentation on enslaved black women.^15^ Collective life experiences and distrust from decades of maltreatment from the medical system, especially in obstetrics and gynecologic care, foster hesitance to seek health care during pregnancy and the postpartum period. In addition, experiencing these inequities throughout a person's life, and especially during pregnancy, can contribute to the concept of “weathering,” which produces physiologic detriments due to the racism and discrimination of racial and ethnic minorities who are also low-income.^16^

Minority pregnant patients, especially those who are low-income and publicly insured, need access to improved, tailored approaches to perinatal care (PNC) that mitigate these social and structural barriers, which drive much of the underlying causes of maternal mortality and morbidity. Setting a new standard of how clinicians approach care is necessary to improve pregnancy outcomes. Current interventions that aim to improve experiences for minority patients receiving PNC are a good start to address the inequities, but must be improved upon to reduce the disparity in mortality and morbidity.^17^ A review of minority pregnant patients' experiences during PNC is necessary to best inform an intervention that improves PNC for this population. While previous reviews have focused on the PNC experience of all pregnant patients, or on the quantitative-based studies of NHB pregnant patients' PNC, we aim to complete a review that highlights the qualitative, narrative-based literature to capture a more in-depth perspective of racial and ethnic minority pregnant patients, with low-income experiences, with their PNC clinicians.^18,19^

The objectives of this scoping review are twofold. First, we aim to investigate qualitative literature describing the experiences of racial and ethnic minority pregnant patients with low income, with PNC clinicians in the United States, focusing on NHB patients. Second, we aim to use this qualitative literature to highlight categories of recurring themes to identify ways that the PNC experience can be improved for racial and ethnic minority pregnant patients with low income.

## Methods

The methodology for this scoping review followed the guidelines set by Askey and O'Malley.^20^ We designed the research question, “What are the experiences of racial and ethnic minority pregnant patients with low income with healthcare clinicians and staff during PNC?” and subsequent search strategy using the SPIDER tool. The SPIDER tool, or sample, phenomenon of interest, design, evaluation, and research type, was used to capture qualitative and mixed methods articles.^21^

The databases used for this review were PubMed, CINAHL, and Web of Science. Search terms were selected to generate an overview of minority patients' experiences with clinicians during PNC (Table 1). While we recognize that minority experiences may be varied depending on each race or ethnicity, we aimed to capture all the articles that depicted qualitative experiences of racial and ethnic minority patients who are low income with the worst perinatal outcomes. Minority was defined as black, Hispanic, Native American, or other racial or ethnic persons who self-identified as being a minority. As such, the search terms represented in Table 1 aimed to reflect our goals. The databases were searched on March 20, 2020, by three authors (D.W., C.C.A., and C.W.) and repeated again on August 19, 2020 to check for any additional article that matched the search criteria. The search was cross-checked to ensure it could be repeated.

**Table 1.** Search String for Each Database for Racial and Ethnic Minority Pregnant Patients with Low-Income Experiences of Perinatal Care

Database	Search string
PUBMED	(((minority OR “african American” OR black OR latina OR hispanic OR “person of color” OR “native American” OR indigenous) (woman OR women OR female))) AND ((“Prenatal care standards”[MeSH] OR perinatal OR prenatal OR postpartum OR maternal) AND (pregnancy[MeSH]) AND ((view^*^[tiab] OR attitude^*^[tiab] OR experience^*^[tiab] OR opinion^*^[tiab] OR perspective^*^[tiab] OR perception^*^[tiab]) OR (barrier^*^[tiab] OR facilitator^*^[tiab])) AND (poverty[MeSH] OR “urban population”[MeSH] OR “low income” OR “inner city” OR medicaid)
WEB OF SCIENCE	(minority OR “african American” OR latina OR hispanic OR “person of color” OR “native american” OR indigenous) AND (woman OR women OR female) AND (perinatal OR prenatal OR postpartum OR maternal) AND (pregnant OR pregnancy) AND ((view OR attitude OR experience OR opinion OR perspective) OR (barrier OR facilitator)) AND (poverty OR “urban population” OR “low income” OR “inner city” OR medicaid)
CINAHL	(minority OR “african American” OR latina OR hispanic OR “person of color” OR “native american” OR indigenous) AND (woman OR women OR female) AND (perinatal OR prenatal OR postpartum OR maternal) AND (pregnant OR pregnancy) AND ((view OR attitude OR experience OR opinion OR perspective) OR (barrier OR facilitator)) AND (poverty OR “urban population” OR “low income” OR “inner city” OR medicaid)

Table of search string used for each database, PubMed, Web of Science, and CINAHL, for low-income minority pregnant patients' experiences with perinatal care providers.

Search results from the three databases yielded 1008 initial results. All citations were imported into the Endnote software to remove duplicates. Once duplicates were removed, 752 articles remained. Articles were then further sorted by title and abstract according to the following eligibility criteria:

(1) Narratives of racial and ethnic minority pregnant patients with low income experiences with PNC clinicians
(2) Studies took place in the United States and are in English
(3) Published from 1990 to present
(4) Studies that utilized an open-ended qualitative methodology (i.e., focus groups, interviews, etc.)

**FIG. 1.**
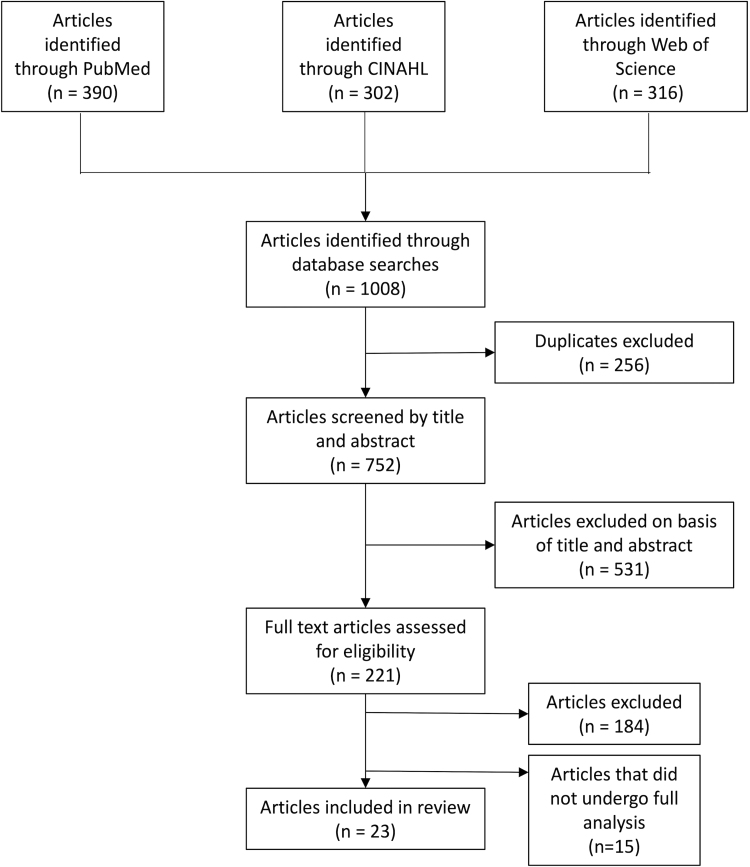
Flowchart of PRIMSA Diagram. PRIMSA 2009 flowchart showing search results through August 19, 2020, and the sequential study selection process. CINAHL, Cumulative Index to Nursing and Allied Health Care.

After sorting by title and abstract, 221 articles were identified for full text review and were organized in Microsoft Excel. Data abstracted from the full-text articles included the following: journal, location, study design, study objectives, conceptual framework, sample size, population demographics, study instrument used, PNC uptake facilitators and barriers, study results, study recommendations, and study limitations. Disagreements were resolved by discussion with the study team. After a full text review, 37 articles satisfied all the inclusion. Articles were then further sorted for quality and relevance by purposeful sampling. The acronym CART (completeness, accuracy, relevance, and timeliness) was used to evaluate the 40 articles, and finally, 23 articles were included for analysis (Table 2 and Fig. 1).^22^

**Table 2.** All Included Articles from Search Results of Literature Review: Racial and Ethnic Minority Pregnant Patients with Low-Income Experiences of Perinatal Care

Title	First author	Year published	Location	Objectives/purpose	Race/ethnicity	Themes
Barriers to service use for postpartum depression symptoms among low-income minority mothers in the United States	Abrams, LS	2009	Los Angeles, California; Newark, New Jersey	Barriers to postpartum depression care.	Latina, black	Communication connection trust
Excess gestational weight gain in low-income and obese women: a qualitative study	Anderson, CK	2015	Madison, Wisconsin	Facilitators to perinatal care, including peer support groups.	Black, Non-Hispanic white, Hispanic	Education communication trust
‘Breaking it down’: Patient-clinician communication and prenatal care among African American women of low and high literacy	Bennet, I	2006	Pennsylvania	Barriers and facilitators such as empathy and effective communication.	Black	Connection education support
Black non-Hispanic mothers' perceptions about the promotion of infant-feeding methods by nurses and physicians	Cricco-Lizza, R	2006	New York	Promotion of infant feeding methods by nurses and physicians.	Black	Support education communication connection trust
Attitudes and perceptions related to smoking among pregnant and postpartum women in a low-income, multiethnic setting	Dunn, CL	1998	Did not specify	Connection to physicians and its relation to reduction of smoking during pregnancy.	Black, Native American, white	Support education connection trust
Support for young black urban women after perinatal loss	Fensternmacher, KH	2019	Pennsylvania	Support/empathy during perinatal loss and lack of education among adolescent mothers.	Black	Support
Anguish, yearning, and identity: toward a better understanding of the pregnant Hispanic woman's prenatal care experience	Fitzgerald, EM	2016	Louisville, Kentucky	Lack of support, language barriers, and frustrations with providers. Also, lack of education and receiving education resources.	Hispanic	Support education communication connection trust
Going beyond the call of doula: a grounded theory of analysis of the diverse roles community-based doulas play in the lives of pregnant and parenting adolescent mothers	Gentry, QM	2010	Georgia	Doula support for young Latina mothers.	Latina	Support education
Barriers to intervention among women experiencing intimate partner violence proximal to pregnancy	Hassalle, K	2020	Did not specify	Barriers and facilitators in IPV care.	Black, Asian American, Hispanic, white	Communication, connection
Qualitative comparison of women's perspectives on the functions and benefits of group and individual prenatal care	Heberlein, EC	2016	Greenville, South Carolina	Empathy, support, and education from clinicians.	Black, white	Support education communication connection trust
The value of a learner's stance: lessons learned from pregnant and parenting women	Humbert, L	2009	Marion County and Lake County, Indiana	Facilitators to perinatal care.	Black, white, Latina	Support communication connection trust
Women's perceptions of outcomes of prenatal case management	Issel, LM	2000	Texas	Barriers and facilitators to perinatal care.	White, Hispanic, black	Support education trust
Barriers to seeking help and treatment suggestions for prenatal depressive symptoms: focus groups with rural low-income women	Jesse, DE	2008	Did not specify	Barriers to sharing depressive symptoms with health care providers.	Black, Caucasian	Support education connection trust
Barriers to prenatal care for Mexican and Mexican American women	Kalofonos, I	1999	San Diego, California	Political economics and sociocultural barriers to use of prenatal care services.	Hispanic (Mexican American)	Support Education Communication Connection Trust
Perinatal loss in low-income African American parents	Kavanaugh, K	2005	Chicago, Illinois	Perinatal loss, lack of connection with providers, empathy, and support.	Black	Connection Support Trust
Having our say: African American and Latina mothers provide recommendations to health and mental health providers working with new mothers living with postpartum depression	Keefe, RH	2016	Rochester, New York	Experienced postpartum depression barriers.	Black, Hispanic	Support Communication Connection
Introduction of Centering Pregnancy in a public health clinic	Klima, C	2009	Chicago, Illinois	Barriers and facilitators to during perinatal care.	Black	Support Education Communication Connection Trust
A will without a way: barriers and facilitators to exercise during pregnancy of low-income, African American women	Krans, EE	2011	Pittsburg, Pennsylvania	Barriers and facilitators to exercise during pregnancy.	Black	Connection Education
Perceptions of mental health services among low-income, perinatal African American women	Leis, JA	2011	Baltimore, Maryland	Mental health services as a barrier to service use among low-income, urban, perinatal African American clients.	Black	Support Communication Connection Trust
Patient-provider communication and counseling about gestational weight gain and physical activity: a qualitative study of the perceptions and experiences of Latinas pregnant with their first child	Lindsay, AC	2017	Massachusetts, Rhode Island	Lack of education where women felt that providers did not give adequate information.	Hispanic	Connection Education Communication
Does Centering Pregnancy group prenatal care affect the birth experience of underserved women? A mixed methods analysis	Liu, R	2017	San Francisco, California	Lack of connection with multiple anonymous providers and lack of empathy response of providers during care.	Hispanic, black, Indian/Alaska Native, white	Connection Education Trust
Anatomy of good prenatal care: perspectives of low-income African-American women on barriers and facilitators to prenatal care	Mazul, MC	2017	Milwaukee, Wisconsin	Barriers and facilitators to receiving PNC in an urban setting	Black	Support Education Communication Connection
Perceptions about prenatal care: views of urban vulnerable groups	Milligan, R	2002	Washington, District of Columbia	Barriers and facilitators to perinatal care.	Black	Connection Trust
Women's experiences of group prenatal care	Novick, G	2011	Did not specify	Support of group prenatal care.	Black, Hispanic	Support Education Communication Connection Trust
The intersection of everyday life and group prenatal care for women in two urban clinics	Novick, G	2012	Did not specify	Facilitators of group prenatal care.	Black, Hispanic	Support Education Connection
Vigilance in parents' experience of fetal and infant loss	Nowak, E	2011	Racine, Wisconsin	Fetal and infant loss and lack of support from providers.	Black, white, Hispanic	Support Communication
Health care experiences of low-income women with prior gestational diabetes	Oza-Frank, R	2018	Ohio	Community coaches providing GDM education and support.	Black, white, Hispanic	Support Education Communication Connection Trust
Understanding perspectives of African American Medicaid-insured women in the process of perinatal care: an opportunity for systems improvement	Roman, L	2017	Michigan	Medicaid-insured women and the barriers involved with their care.	African American	Support Education Communication Connection
You learn to go last: perceptions of prenatal care experiences among African American women with limited incomes	Salm Ward, T	2013	Milwaukee, Wisconsin	Barriers of racial discrimination during perinatal care.	African American	Communication
Low-income, urban minority women's perceptions of self-care and infant care during the postpartum period	Suplee, P	2014	Did not specify	Barriers of self-care and infant care during the first 6 months postpartum.	Black, Hispanic	Education
Understanding low-income African American women's expectations, preferences, and priorities in prenatal care	Tucker Edmonds, B	2015	Philadelphia, Pennsylvania	Prenatal care attendance and preference for prenatal care experiences.	Black	Support Education Communication Connection Trust
Intimate partner violence during the perinatal period	Wadsworth, P	2018	Did not specify	Facilitators to IPV during perinatal period.	Black, white	Support Education Communication Connection
Women's narratives on quality in prenatal care: a multicultural perspective	Wheatley, R	2008	Chicago, Illinois	Midwives and lack of empathy and connection with patients.	Black, Hispanic (Mexican American, Puerto Rican), white	Support Education Communication Connection Trust
Clients' perceptions of the value of prenatal psychosocial services	Wilkinson, DS	1999	California	Psychosocial services and barriers to care.	Black, Latina, white	Support Connection
Barriers and facilitators to recurrent preterm birth prevention among low-income women: a qualitative study	Yee, LM	2019	Chicago, Illinois	Barriers and facilitators of initiation of psychosocial services.	Black, Hispanic, white	Education Communication Connection Trust
Perceptions of coercion, discrimination, and other negative experiences in postpartum contraceptive counseling for low-income minority women	Yee, LM	2011	Chicago, Illinois	Insufficient counseling and lack of education	Black, Hispanic	Education Communication Connection Trust
Development of prenatal event history calendar for black women	Yi, CH	2008	Did not specify	Providers connecting by overreaching.	Black	Connection

Table of all 38 included articles on pregnant patients' experiences with perinatal care providers. This includes articles that met all the inclusion and none of the exclusion criteria.

Thematic synthesis was conducted through line-by-line coding with ATLAS.ti research software. This allowed for a systematic and critical analysis of the diverse sources, while minimizing inaccuracy and bias.^23^ All codes were inductively developed during an initial and secondary reading of the articles. Coding was then reviewed by a second author to ensure agreement. Next, codes were examined to develop groupings and uncover themes. Codes with three or more quotes linked were included.

This review involved publicly available literature and de-identified patient information. As such, Institutional Review Board approval and informed consent were not necessary.

## Results

**FIG. 2.**
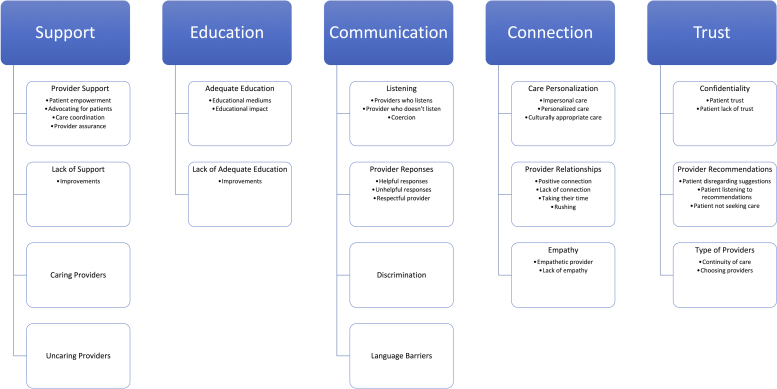
Thematic Synthesis Coding Tree for Low-Income, Minority Patients' Experiences with Perinatal Care. Thematic categories, themes, and subthemes of low-income, minority patients' experiences with perinatal care. The five main categories seen in the literature were that of education, support, communication, connection, and trust.

Of the 23 final articles, 22 studies were purely qualitative, and one was mixed methods. All included the perinatal experiences of minority pregnant patients with clinicians or health care staff. Findings from 23 studies were synthesized into five analytical categories, which represent an interpretative analysis of various experiences in PNC. Five major categories emerged: support, education, communication, connection, and trust (Fig. 2 and Table 3). These categories are discussed in terms of what pregnant patients narrated during PNC, observations, reactions, and preferences relating to each theme.

**Table 3.** Illustrative Quotes of Racial and Ethnic Minority Pregnant Patients with Low-Income Experiences of Perinatal Care

Themes	
Support	
Provider support	“I think good prenatal care is like when I would miss they would call me,…ask me if I was ok and tell me how important my visits are…they want to make sure the baby is ok.”^34^
Lack of support	“Because then I come in looking and asking, and you [the provider] shoot me down and make me feel like I didn't get nothing out of you”^37^
Caring providers	*After suffering infant loss*: “The people in the hospital were all very generous and helpful. They were very sympathetic. They expressed their condolences and everything. So that's another part that helped”^25^
Uncaring providers	“What's the point of going? She [provider] don't care…she don't have to care either, (laughing) but it'd be nice to pretend”^34^
Education	
Adequate education	“I had nurse-midwives…every single appointment she would just come and sit with me. Any question I had to ask her she would just sit there and answer all of my questions.”^40^
Lack of adequate education	“I don't know what you all are saying. I mean, I'm like I don't know what you all are talking about (patient with fibroid tumors). I don't know what you all are saying”^37^
Communication	
Listening	*When discussing nurses:* “Their experience, the way they connected to us, they didn't just hear us, they listened to us. They took it upon themselves to take it a step further when we needed that extra…We were human”^38^
Provider responses	“I asked the doctor about back pains, and he laughed and said, You're pregnant! I was offended.”^29^
Discrimination	“I don't really speak the language, so I just keep quiet, but it really bothers me because sometimes you go with an emergency or a pain or something and they are really angry. They aren't nice in some clinics. It's awful because I feel personally discriminated against because if someone goes in with an emergency with pain and they have an angry face when they are with you…You know I understand a little, and they are laughing and talking, and I know because they say, “Spanish” and I ask why are they like that?”^26^
Language barriers	“I'm worried about the day I go into labor, will there be an interpreter there for me? Or should I look for someone who will translate for me so I don't have to fight for it, or how should I do it honestly that really makes me anxious”^26^
Connection	
Care personalization	“She just came in and it's like she is so used to it and you are just another pregnancy, so she doesn't see you for an individual. She just does her think and leaves”^37^
Provider relationships	“I can pretty much tell her any issue that I'm having or any questions that I have and I mean, she's totally-I mean, it's like I'm talking more to a friend who knows about it, than a doctor who tells you what you should feel and all this other stuff, they explain everything to you when you're like, ‘Why is this doing this? I've never heard of that? They'll explain it to you. It talks a lot of the stress away.”^28^
Empathy	“Just letting them know it's normal, it's natural and you're going to have some feelings of doubt or some feelings that you may not be comfortable with. It takes a while for people to really accept the fact that they are actually pregnant and for it to become real to them. And I think that them knowing that their feelings may not be abnormal.”^31^
Trust	
Confidentiality	“Doctors always say its going to be kept confidential. Then they compare to other doctors and stuff like that. It gets on my nerves sometimes when another doctor comes and asks about something that he shouldn't even know about. I guess you can trust them one time, but when they do you wrong you never trust again. I mean you gonna have to take chances”^31^
Provider recommendations	*On discontinuing medications recommended:* “And I said, “Well why are you giving it to me?” And she [the provider] said, “So you can go to sleep.” She said “If you're asleep, you ain't depressed.” I Said, “I've got children, I can't sleep all day how can I take care of my children when I'm sleeping?” So I stopped taking the medication”
Type of providers	“Student doctors came in and out of the room all the time, disturbing me and waking me up. They practice on you like you're a guinea pig”^29^

Quotations of the different categories and subcategories represented in this review article on minority pregnant patients' experiences with perinatal care.

## Categories with Recurring Themes

### Support

Eighteen of the 23 articles described pregnant patients' perceptions of having supportive staff members while receiving care.^24–41^ The category of supportive staff included perceived adequacy of support from clinicians. Subthemes of adequacy of supportive staff included the following: empowering patients, reassuring patients, clinicians care coordination, and suggested improvements. Furthermore, support was the most coded category, suggesting the importance of support in the PNC experience.

#### Adequate clinician support

Fifteen articles discussed adequate support from clinicians and staff.^24–32,34,36,37,39–41^ Supportive staff generally provided both emotional support and tangible perinatal resources. Nine articles discussed clinicians being supportive by empowering patients to engage in self-care and self-education during pregnancy.^24,26,28,30,32,35–38^ Patients wanted to engage actively and participate in their care and appreciated clinicians who gave them ownership over their care. Many articles described patients expressing that they felt they would get the most benefit out of a patient-provider interaction if the provider empowered them and gave them autonomy by presenting a complete and holistic picture of their current health status.^26,28,35^ Eight articles discussed clinicians showing support by reassuring patients, including notifying the patients about the status of their baby's health.^28,30–34,37,41^

Three articles discussed how patients sought encouragement from clinicians.^24,25,28^ Supportive teams helped patients overcome fears, while providing a supportive environment for pregnant patients to feel at ease and ask questions.^28^ One prevalent example of support was actually represented by groups that are considered non-clinicians, who helped patients during their PNC. In the article by Gentry et al., doulas were said to offer extensive support, often with patients relying on them to get through their pregnancies. Undocumented immigrant patients in this study described doulas offering help to them by finding resources, providing transportation for visits, and advocating for the patients' birth decisions.^27^

#### Lack of clinical care team support

Seven articles discussed lack of clinician support.^24,26,28,34,36,37,41^ Of the seven articles, two cited that pregnant patients felt clinicians rushed during their prenatal care visits.^24,34^ Another two articles discussed that patients often felt they did not receive consistent support from clinicians and felt lost throughout their pregnancies.^26,36^ Other concerns were that clinicians did not adequately address pregnancy complications^28^ and did not inform patients of outside services.^36^ Clinicians' inflexibility to help patients change their appointment times was also cited.^37^ Two of the seven articles mentioned improvements for lack of support during PNC.^32,39^ These noted that clinicians should refer patients to additional community resources, especially for those patients experiencing homelessness^32^ and IPV during pregnancies.^39^

### Education

The category of education was discussed in 16 of the 23 studies reviewed and had one of the most coded findings.^24–28,30,31,34,36–40,42,43^ The theme of education was “adequacy.” Subthemes included the following: adequate and lack of adequate education, different educational mediums, and improvements for education.

#### Receiving adequate education from clinicians

Eleven articles reported the subtheme of adequate education during their PNC.^24,25,27,28,30,31,34,36,37,39,40^ Education was deemed adequate when patients felt that they received enough information throughout their PNC to feel comfortable or confident with their pregnancy, delivery, or postpartum self-care and infant care. Of the 11 articles, four discussed receiving adequate professional guidance on perinatal techniques and/or health conditions.^24,25,27,36^ Gentry et al. reported that younger patients who felt frustrated about not knowing what to do with their babies expressed that their doulas were crucial in modeling how to have maternal-infant bonding and productive ways of soothing comforting newborns.^27^ Two of the 11 articles discussed other examples of receiving adequate education in the context of postpartum care, such as birth control counseling after birth.^27,28^

Varying media to deliver education were also identified.^24,28,30,31,36,39^ Written pamphlets, for example, were deemed helpful as supplemental information during a study by Issel.^30^ In addition, an article by Brown et al., which was not included for thematic analysis, discussed the usage of cell phone and text messages to deliver pertinent health information.^44^ Finally, education was perceived as adequate when women were given sufficient opportunity to ask questions and clinicians gave detailed responses.^28,37,40^

#### Lack of adequate education from clinicians

Eight articles reported the subtheme of inadequate PNC education.^24,26,28,34,36,37,40,43^ Education was “inadequate” when patients felt they did not receive enough information throughout the various stages of pregnancy, especially postpartum. The lack of knowledge was often associated with feeling unprepared and frustrated. Of the eight articles, five discussed how patients wish they received more training to feel more comfortable with their pregnancies.^24,26,28,36,37^ For example, in the article by Cricco-Lizza, many patients expressed lack of satisfaction in breastfeeding education where patients left the hospital feeling uncomfortable and unprepared.^24^ In addition, study participants noted that they had never received information about infant feeding from any of their providers at any point during pregnancy and postpartum.^24^

Three studies discussed difficulties with inadequate education and clinicians lacking time to educate patients during perinatal visits.^34,36,37^ Questions were often not answered, and patients felt they left the visit with little to no information.^37^ Time constraints during medical visits sometimes led to clinicians giving medical explanations that were brief, and too difficult to understand. A study by Oza-Frank et al. expressed the notion that pregnant patients with gestational diabetes (GDM) would have changed their care seeking habits, such as going to follow-up appointments, if they had received more education about the risks of GDM.^36^ In addition, the study by Fitzgerald et al. discussed how low-income Hispanic patients sometimes felt they could not speak up during visits due to not speaking English, which left many feeling as though they did not receive adequate care.^26^ Finally, two articles discussed lack of proper education and some educational techniques as harmful.^28,39^ For example, in the article by Wadsworth et al., patients reported clinicians and staff should be more cautious of how intimate partner violence (IPV) is addressed, as pregnant patients may suffer consequences from an abusive partner if a patient brought home information such as pamphlets regarding IPV.^39^ Finally, five articles suggested methods for improvements such as tailoring care plans and appointment times to reflect the needs of patients, and using more technology resources to communicate with patients through different mediums.^32,36,38,40^

### Communication

Seventeen of the 23 articles discussed communication in PNC.^24,26,28,29,32–40,42,43,45,46^ Themes of communication included clinicians listening and not listening to patients, disregarding health concerns, coercion, helpful clinician's responses, respecting patients, discrimination and racism, and language barriers.

#### Listening

Fourteen articles discussed the topic of how well clinicians listened to their patients during PNC.^24,28,29,32–35,37–40,42,43,45^ Of these, two described the “ideal” listener as someone who did not judge, was warm and empathetic, and had excellent listening skills.^24,45^ Pregnant patients also valued clinicians who considered their opinions,^29^ took their time,^32^ and made sure patients left the visits with good explanations and confidence about their pregnancy.^40^

In addition, seven of the 14 articles discussed patients feeling their clinicians did not listen to them.^24,33,34,37,38,40,43^ In these articles, pregnant patients often felt uncared for and rushed,^34^ repeated themselves continuously as if no one was listening,^37^ and felt less than satisfied with clinician's listening skills.^40^ Other examples included patients' dissatisfaction with changing physicians due to multiphysician practices, which in some cases lead to discontinuing of service altogether.^24,33^

Seven of the 14 articles discussed clinicians disregard of patient concerns.^28,29,35,37,45^ These included patients feeling dismissed during encounters and discouraged from asking clinicians questions.^28,37,39,45^ The article by Abrams et al. mentioned one patient who told her clinician about severe postpartum depression and suicidal ideations was dismissed and told to return in 6 weeks.^45^ Finally, in the article by Yee and Simon, patients tried to be heard by coercive and overbearing clinicians that they failed to appreciate their reproductive health choices.^43^

#### Clinician responses to patients

Ten of the 17 articles discussed clinician's responses to patients.^24,28,29,33,34,37,40,42,43,45^ Of these, seven described clinician responses as unhelpful.^24,29,33,37,40,43,45^ These were often associated with patient's attempts to seek help and clinicians not fully answering questions or outrightly dismissing questions.^24,29,33,40,45^ Three articles discussed clinician responses as being helpful.^28,33,42^ These included clinicians giving practical advice and patients feeling like they had necessary recommendations made in their care.^28,33,42^ Four articles discussed clinicians being respectful.^24,29,34,40^ Two of these four articles stressed that respectful and dignified treatment by health care professionals seemed more critical than logistical factors.^30,40^

#### Discrimination and racism

Seven of the 17 articles touched on discrimination and/or racism.^29,34,36,37,40,43,46^ Perceived racism and discrimination practices included tracking by ethnicity, lack of bilingual staff, cultural incompetence, and getting talked down to or mistreated because of age, socioeconomic status, or race. Most of the articles that discussed discrimination were about discrimination due to income level, and not necessarily race. Socioeconomic status was often reflected in clinicians being judgmental or patients perceiving they received different treatment because of having Medicaid insurance.^37,40,46^ Only three articles directly discussed women feeling like they were being treated differently, or worse, because of their race.^26,43,46^

#### Language barriers

Two articles discussed language barriers.^26,29^ These included worrying and fighting to get an interpreter during care. Patients worried about their medical care when they did not speak English, and they worried about being judged or misunderstood because they were foreigners. These patients emphasized the need for culturally appropriate services.^26,27^

### Connection

Eighteen of the 23 articles discussed connection in PNC.^24–26,28,29,31–34,36–41,43,45,47^ Components of connection included care personalization, clinician relationship, and empathy.

#### Care personalization

Eight of the 18 articles discussed impersonal care^24,26,28,32,36,37,40,43^ These included patients distrusting the impersonal or insensitive care from clinicians who treated them coldly or in an unfriendly manner.^24^ Patients also preferred attentiveness and availability in PNC encounters and were dissatisfied with physicians who were absent from clinic visits or offered impersonal counseling.^40,43^ Three of the eight articles discussed personalized care that was culturally appropriate, where clinicians used less medical jargon and allowed for more comprehendible and reciprocal dialog.^24,28,36^

#### Clinician relationship

Fourteen of the 18 articles discussed relationships with clinicians.^24,26,28,29,31–34,37–41,45^ Of these, seven mentioned positive clinician connections.^28,29,31,32,38,39,45^ Two articles discussed a desire to connect with clinicians through repeated clinical visits with the same team. These positive and long-term relationships were often vital to success.^32,45^ Pregnant patients also preferred clinicians who remembered their faces and tried to form bonds with them as patients.^29,31,38^

Eight of the 18 articles discussed the lack of connection during PNC.^26,29,31,33,38,40,41,45^ These included patients feeling as though the clinician was not trying to get to know them, wanting more culturally appropriate services, or not feeling that they could relate to clinicians.^26,31,33,45^ Patients wanted to feel connected and special, but were unable to because of the lack of connection with their clinical care providers.^38^ The article by Wilkinson and Gonzalez-Calvo also discussed patients' feelings of being misunderstood and judged for prior life decisions such as substance abuse.^41^

#### Clinicians who take their time

Six of the 18 articles discussed clinicians taking their time with patients.^24,28,29,32,34,40^ This included clinicians answering questions thoroughly and spending enough time with the patient.^24,40^ Three articles discussed clinicians rushing during encounters.^32,33,37^ Pregnant patients disliked having to wait and then be hurried through a quick examination, and expressed overall dissatisfaction with their care.^37^

#### Empathy

Eight of the 18 articles discussed empathy.^24,25,31,32,37,39,43,47^ Empathetic clinicians were those who understood concerns, helped with fears and uncertainty, and were helpful and generous.^24,25,31,32^ Two of the eight articles discussed lack of empathy as not considering personal barriers, blaming patients for things they had no control over, and making uncomfortable situations more difficult.^37,43^ Finally, three articles discussed the need for improving empathy in PNC.^32,39,47^ This included clinicians needing to be more understanding and patient when patients disclose such personal issues as abuse and IPV.^39^

### Trust

Seventeen of the 23 articles discussed trust.^24–26,28–31,33,36–38,40,42,43,45,47^ Trust was further broken down into the themes of concerns about confidentiality, trust in clinicians' recommendations, and types of clinicians who impact level of trust.

#### Confidentiality

The theme of confidentiality was discussed in six articles.^24,31,33,36,37,47^ Lack of trust in the ability of clinicians to maintain confidentiality was expressed in three of the six articles.^31,33,47^ This was noted by Jesse et al., where patients suffering from depression felt that clinicians breaking confidentiality and sharing the patients' personal information was a major barrier in their willingness to seek the care.^31^ In addition, patients also expressed fear that disclosing information and seeking help would result in clinicians judging them as incompetent and unable to care for their child.^36,37^ In the article by Roman et al., examples were given of patients who did not enroll in social support programs to not come across as if they could not care for their child.^37^

#### Clinician recommendations

Trust, or lack thereof, in clinician recommendations was expressed in six articles.^24,28,30,37,40,43^ Three articles discussed patients who did not want to listen to clinician's recommendations because they felt that the information they received was incorrect.^24,37,40^ Another reason cited for mistrust was seen in the article by Yee and Simon where patients described being hesitant to listen to clinicians' recommendations on birth control prescribed postpartum because they suspected the clinician may be benefiting financially from the prescription.^43^ Three of the six articles described patients who trusted their clinician's recommendations.^24,28,30^ Reasons cited were either the clinician taking their time to fully explain recommendations and procedures or patients feeling that they had complete access to their clinicians.

Finally, the type of clinician played a major role in patients expressing trust. Caregiver preference was expressed in 12 articles.^24,25,27–29,31,32,36–38,40,42^ Clinicians who patients trusted most were community health workers, midwives, and doulas because they seemed relatable to patients.^27,37,40^ In addition, maintaining the same clinicians and having continuity of care impacted trust with many patients wanting to maintain the same clinician.^25,28,29,31,32,38^

## Discussion

Understanding why deep racial inequities exist in the U.S. maternal mortality rate is critical for developing appropriate target interventions and programs. Organizations such as the American College of Obstetricians and Gynecologist (ACOG), Merck for Mothers, NIH Improve Initiative, Maternal Health Hub, and various state and local public health departments have started important work on the impact of social determinants of health and racism on maternal mortality.^48–52^ Qualitative studies that capture patients' PNC experiences are critical to elevating minority patients' voices to ensure that interventions and programs to ameliorate these egregious inequities are developed correctly. This review highlights the experiences of racial and ethnic minority pregnant patients with low income and presents the major categories encompassing the need for support, education, connection, communication, and trust during PNC.

The lack of articles directly addressing the experience of racism in PNC was notable. Only two of the 23 articles included directly expressed patients' feelings of discrimination directly tied to their race. Racism belies many of the inequities that drive social, structural, economic, and political factors that promulgate maternal health inequities. The absence of racism being addressed as a root cause of such maternal health inequities is an important finding in this scoping review and merits attention. If interventions are to be designed to address minority differences in severe maternal morbidity and maternal mortality outcomes, racism should be addressed as a root cause.

In addition, it should be noted that although the prevailing themes in this review are discussed separately, many of them intersected. The category of trust, for example, intersected with adequate communication and ability to receive education from clinicians. This review and the literature have shown that trust is critical in reducing health inequities among minority patients.^53–55^ Distrust in the health care system has led to patients not seeking or adhering to clinical care recommendations and building a relationship with the clinician has been shown to increase a patient's willingness to follow the clinician's guidelines and recommendations.^56^ As interventions are developed for racial and ethnic minority pregnant patients with low income in PNC, these patients' experiences must be integrated to support increased patient adherence to care recommendation. Improved collaboration between patient and clinician may lead to an overall improved experience of care.

Clinician support was a highly emphasized theme in this review, with care coordination being a critical component of support. Care coordination involves recommendations and tailoring support services.^57^ Studies have shown that PNC coordination impacts care utilization, and may also improve health outcomes.^58–60^ While more studies are needed to elucidate what method of care coordination works best in varying health care settings, policymakers and leaders should examine ways to implement care coordination as a stable presence in PNC, especially in low-income communities.

Finally, in our review, communication played a major role in patient's feeling heard and satisfied with the care they received. How clinicians chose to communicate also impacted the patient's level of trust in their clinicians. As in our study, a systematic review by Janssen and Lagro-Janssen found that patient satisfaction and comfort in their obstetric care increased when clinicians used patient-centered communication styles.^61^ This can be defined as working to understand the patients' viewpoints and unique cultural context from which the patients come, and working in collaboration with the patient to find solutions.^62^ Clinicians should aim to tailor their communication style to the patient during each visit, and training can give clinicians the tools to communicate more effectively with patients, especially those from different races, ethnicities, socioeconomic statuses, or backgrounds.

Several limitations exist within this study. First, the search terms and search strategy may not be adequate to encompass all the existing articles on the experiences of racial and ethnic minority pregnant patients, with low income, with PNC. In addition, while thematic synthesis is a widely used method for creating themes in qualitative reviews, there are biases and personal assumptions introduced during the development of these themes. We attempted to mitigate this by reflexivity and evaluating how our biases introduce subjectivity.^63^ Despite these limitations, this review provides a strong foundation to explore experience of racial and ethnic minority pregnant patients, with low income, within PNC and an overview for those designing interventions in this area.

## Conclusion

This review captured prevailing themes in the existing literature on racial and ethnic minority pregnant patients with low income and their experiences with PNC and their clinicians. These qualitative studies provided specific examples of what low-income patients deemed positive and negative during the perinatal period and outlines ways that these experiences can be improved. Future studies can leverage the cited experiences from this review to help inform intervention design to mitigate the negative experiences and health outcomes that minority pregnant patients have in the perinatal period. Finally, future studies should also address gaps in the literature by studying minority pregnant patients' experiences by race or ethnicity, versus grouping them together as monolith of people. By doing this, interventions can be designed to better address some of the health disparities that each group faces and will be an important step in alleviating some of the health disparities in maternal care.

## Abbreviations Used

CINAHL: cumulative index to nursing and allied health care
GDM: gestational diabetes
IPV: intimate partner violence
MMR: maternal mortality ratio
NHB: non-Hispanic black
NHW: non-Hispanic white
PNC: perinatal care
